# Correction: Changing lifestyle for dementia risk reduction: Inductive content analysis of a national UK survey

**DOI:** 10.1371/journal.pone.0244399

**Published:** 2020-12-16

**Authors:** Alessandro Bosco, Katy A. Jones, Claudio Di Lorito, Blossom C. M. Stephan, Martin Orrell, Deborah Oliveira

In the Methods section, a reference is omitted from the second sentence of the first paragraph and the Nottingham Research Ethics Committee project ID is omitted from the third sentence of the first paragraph. The correct sentences are: Full details of the survey methods are published elsewhere (Oliveira et al., 2019). This study received ethical approval from the East Midlands—Nottingham Research Ethics Committee (IRAS project ID 177280; REC reference 16/EM/0044).

In the Discussion section, the same reference is omitted from the first sentence of the third paragraph.

The correct sentence is: Our previous survey results looking at willingness to reduce alcohol intake for dementia risk reduction (Oliveira et al., 2019) showed a positive relationship between having a healthy lifestyle and willingness to reduce alcohol intake (e.g. engaging in physical activity).

The reference is: Oliveira, D., Jones, K. A., Ogollah, R., Ozupek, S., Hogervorst, E., & Orrell, M. (2019). Willingness to Adhere to Current UK Low-Risk Alcohol Guidelines to Potentially Reduce Dementia Risk: A National Survey of People Aged 50 and Over. DOI: 10.3233/JAD-181224.
